# Dendritic Cell-Based Immunotherapy for Myeloid Leukemias

**DOI:** 10.3389/fimmu.2013.00496

**Published:** 2013-12-31

**Authors:** Christian M. Schürch, Carsten Riether, Adrian F. Ochsenbein

**Affiliations:** ^1^Tumor Immunology, Department of Clinical Research, University of Bern, Bern, Switzerland; ^2^Institute of Pathology, University of Bern, Bern, Switzerland; ^3^Department of Medical Oncology, Inselspital, University Hospital Bern, Bern, Switzerland

**Keywords:** dendritic cells, immunotherapy, active, myeloid leukemia, minimal residual disease, leukemia stem cells

## Abstract

Acute and chronic myeloid leukemia (AML, CML) are hematologic malignancies arising from oncogene-transformed hematopoietic stem/progenitor cells known as leukemia stem cells (LSCs). LSCs are selectively resistant to various forms of therapy including irradiation or cytotoxic drugs. The introduction of tyrosine kinase inhibitors has dramatically improved disease outcome in patients with CML. For AML, however, prognosis is still quite dismal. Standard treatments have been established more than 20 years ago with only limited advances ever since. Durable remission is achieved in less than 30% of patients. Minimal residual disease (MRD), reflected by the persistence of LSCs below the detection limit by conventional methods, causes a high rate of disease relapses. Therefore, the ultimate goal in the treatment of myeloid leukemia must be the eradication of LSCs. Active immunotherapy, aiming at the generation of leukemia-specific cytotoxic T cells (CTLs), may represent a powerful approach to target LSCs in the MRD situation. To fully activate CTLs, leukemia antigens have to be successfully captured, processed, and presented by mature dendritic cells (DCs). Myeloid progenitors are a prominent source of DCs under homeostatic conditions, and it is now well established that LSCs and leukemic blasts can give rise to “malignant” DCs. These leukemia-derived DCs can express leukemia antigens and may either induce anti-leukemic T cell responses or favor tolerance to the leukemia, depending on co-stimulatory or -inhibitory molecules and cytokines. This review will concentrate on the role of DCs in myeloid leukemia immunotherapy with a special focus on their generation, application, and function and how they could be improved in order to generate highly effective and specific anti-leukemic CTL responses. In addition, we discuss how DC-based immunotherapy may be successfully integrated into current treatment strategies to promote remission and potentially cure myeloid leukemias.

## Introduction

During the last century our molecular and mechanistic understanding of the immune system and the immunosurveillance of solid and hematological tumors has advanced extensively. For hematological tumors especially, the demonstration of the graft-vs.-leukemia (GvL) effect of allogeneic hematopoietic stem cell transplantation (aHSCT) and donor lymphocyte infusions (DLIs), as well as the discovery of leukemia-associated antigens (LAAs) was of fundamental importance in order to translate, implement, and optimize immunotherapies against myeloid leukemias. Consequently, active and passive immunotherapy approaches, such as peptide- and dendritic cell (DC)-based vaccines using LAAs, monoclonal antibodies (mAbs), and the *in vitro*-generation of leukemia-specific cytotoxic T cells (CTLs) for adoptive transfer have recently yielded promising results in pre-clinical models and clinical trials (1–4). To maximize their efficacy, these immunotherapies have to be implemented into the treatment strategy in conjunction with standard treatments of care for each patient individually. Here, we summarize recent advances in DC-based active vaccination using LAAs and discuss this method as an attractive supplementary immunotherapeutic strategy in the context of current standard treatments for myeloid leukemias.

### Classification, epidemiology, and clinical manifestations of CML and AML

**Figure 1:**
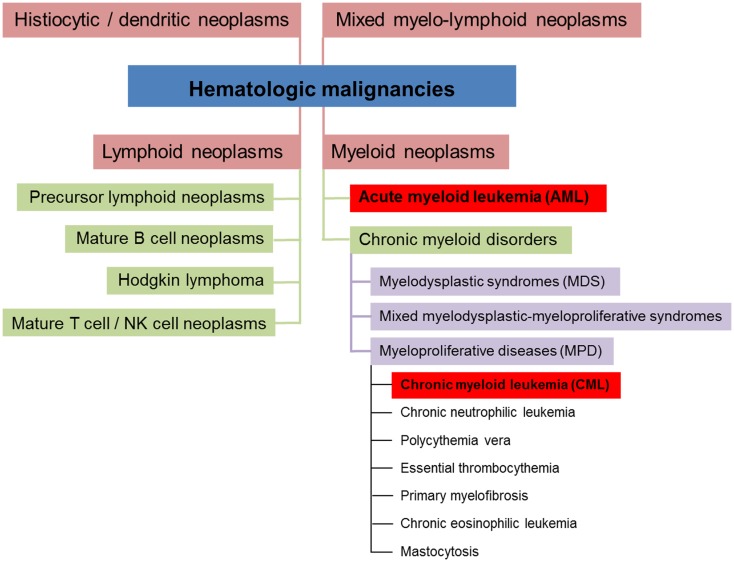
**Conceptual classification of hematologic neoplasms [based on data from Ref. (5, 6)]**.

Hematologic malignancies are neoplasms of the blood-forming system. Conceptually, these neoplasms can be divided into four different subsets (myeloid, lymphoid, mixed myelo-lymphoid, and histiocytic/dendritic neoplasms, see Figure 1) (5, 6). Myeloid neoplasms can be further grouped into acute myeloid leukemia (AML) and chronic myeloid disorders depending on the percentage of bone marrow (BM) infiltration by immature blasts. 20% and more infiltrating immature blasts define the cut-off criterion for AML. Chronic myeloid disorders such as chronic myeloid leukemia (CML) bear the risk of evolving into AML. Experimental studies revealed that myeloid leukemias in general are of clonal origin, suggesting genesis from a single leukemia stem cell [LSC, reviewed in Ref. (7)].

**Figure 2:**
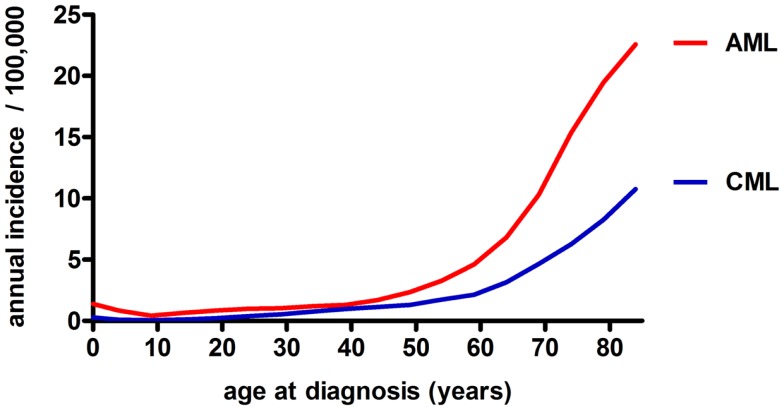
**Annual incidence of AML and CML in the USA among different age groups (both sexes, all race groups, years 1992–2010), based on data from the NCI/SEER (11)**.

Chronic myeloid leukemia is caused by translocation of chromosomes 9 and 22 in a hematopoietic stem cell (HSC) resulting in the formation of the constitutively active tyrosine kinase BCR/ABL1 and the subsequent generation of an LSC (8). CML is characterized by the overproduction and accumulation of mature, functionally impaired myeloid cells, predominantly granulocytes. CML represents about 15–20% of all leukemias in adulthood, affecting slightly more men than women (ratio ~1.8–1) (9). Its annual incidence is 1–2 cases per 100,000 for all age groups (10). This incidence is rising with age to 10–12 cases per 100,000 for people older than 80 years of age (Figure 2) (11). Without treatment, chronic phase CML inevitably evolves via an accelerated phase (12) into blast crisis, which is characterized by the presence of ≥20% blasts in the blood or BM or the presence of extramedullary infiltrating blasts. In two thirds of cases, the blasts are of myeloid origin and the disease phenotype is similar to AML. The other third is of lymphoid origin. Blast crisis CML is highly resistant to treatment, and median survival of patients is approximately 4–8 months. The most common causes of death in blast crisis CML are bleedings and infections due to lack of a functional hematopoietic system (13). Because BCR/ABL1 is necessary and sufficient for the malignant phenotype, attempts to inhibit this kinase using small molecules have led to the discovery of the specific tyrosine kinase inhibitor (TKI) Imatinib (14). Since its introduction into clinics in 2001, Imatinib became the gold standard in CML therapy and has replaced cytarabin/interferon (IFN)-α combination therapy (15). Imatinib is the first-line therapy of choice for nearly all newly diagnosed CML patients (16). Second- and third-generation TKIs with superior efficacy, also against mutated forms of BCR/ABL1, are currently tested in clinical trials (17–20). Even though TKI treatment stabilizes the disease during the chronic phase, a small percentage of patients will progress to accelerated phase and blast crisis (21). Besides TKIs, which have demonstrated long-term disease control and very good tolerability, the only other treatment option that may be considered for CML is aHSCT. Today, aHSCT is the only treatment with proven ability to cure CML (22).

In contrast to CML, AML is an aggressive and fatal disease caused by an increased proliferation and a block in differentiation capacity of myeloid blasts. With an annual incidence of three to five cases per 100,000 (m:f ratio, 3:2), AML is the most frequent myeloid leukemia in adults (10). Compared to CML, the age-dependent rise in AML incidence is even more drastic to peak at 20–23 cases per 100,000 in the geriatric population (Figure 2) (11). Besides age and sex, known risk factors for myeloid leukemias include exposure to ionizing radiation, benzene (e.g., cigarette smoking) and previous cytotoxic chemotherapy (23, 24). Despite the tremendous efforts that have been made to classify AML based on cytogenetic and molecular markers (25), AML treatment basically remained unimproved in the last 20 years and consists of induction cytotoxic chemotherapy (“3 + 7” scheme with cytarabin and an anthracycline), with minor modifications for elderly patients, therapy-related AML, and relapsed or therapy-resistant disease. The only exception is *t*(15;17)-associated acute promyelocytic leukemia (APL), which is treated with a differentiating agent, all-trans retinoic acid (ATRA) in combination with standard chemotherapy (23, 26). In face of these highly toxic chemotherapies, on average less than 30% of AML patients survive long-term. The prognosis for “elderly” patients (defined by the age of 65 or more in most studies) is even more dismal. Treatment failure may occur due to therapy-related complications, such as infections, toxicity, and tumor lysis syndrome. More importantly, the high disease relapse rate after a first remission is the major problem clinicians are confronted with in AML therapy (23). Relapse is thought to be caused by a therapy-resistant neoplastic cell reservoir slumbering in the BM, a situation referred to as minimal residual disease (MRD). It is likely that MRD represents the persistence of quiescent, therapy-resistant LSCs in the BM. Therefore, after a first remission is achieved, post-remission chemotherapy and/or aHSCT is needed to control LSCs (27).

### Leukemia stem cells and the problem of minimal residual disease

The goal of therapy in myeloid leukemia is to induce a durable complete remission (CR). For chronic phase CML, this is most often relatively easily achieved by TKI treatment; however, this therapy only eliminates the bulk of leukemia cells, whereas LSCs are spared. It is thought that CML LSCs are not completely addicted to BCR/ABL1, and several studies have shown survival of CML LSCs in the presence of Imatinib *in vitro* and *in vivo* [(28) and reviewed in Ref. (20)].

For AML, induction poly-chemotherapy may result in a labile CR that has to be consolidated by aHSCT or post-remission chemotherapy. If this treatment is omitted, relapse will often occur rapidly due to persistence of MRD below the cytological detection limit of ~10^9^ cells (23).

Whereas CML LSCs are relatively well characterized as lineage-negative (lin^−^) CD34^+^CD38^−^ cells, the definition of the immunophenotype of AML LSCs is currently controversially discussed. Generally, LSCs are defined as a rare cell population with the capability of self-renewal, extensive proliferation, induction of leukemia, and serial transplantation capacity in xenografts as well as resistance to various treatments. Seminal studies by John Dick et al. using severe combined immunodeficiency (SCID) or non-obese diabetic (NOD)/SCID mice in the 1990s revealed that AML stem cells reside within the lin^−^ CD34^+^ CD38^−^ fraction, as the initiation of AML of all subtypes (except APL) was only possible with purified lin^−^ CD34^+^ CD38^−^ cells, but not with purified lin^−^ CD34^+^ CD38^+^ cells. The leukemias produced in these mouse models closely resembled the original human diseases, providing evidence that AML stem cells have long-term self-renewal capability and determine the leukemia’s phenotype (29, 30). Based on these experiments, the authors hypothesized that leukemias are hierarchically organized in a similar way as the normal blood-forming system and that the normal HSC would most likely be the cell-of-origin that is malignantly transformed during leukemogenesis. Subsequently, many groups tried to refine the immunophenotype of AML LSCs, and several additional markers were characterized (31–36). However, findings from a recent study by Sarry et al. have questioned this strict definition of LSCs by immunophenotype. These authors showed that CD34 expression in AML is highly variable, classifying their patients into 3 groups based on the extent of CD34 expression. Importantly, LSCs were found in all samples, even in CD34 negative ones, and in some patients also in a cell population expressing low amounts of lineage markers. Therefore, these authors suggest that the absolute distribution of LSCs does not necessarily correlate with their phenotypic distribution so that even though LSCs are enriched in certain fractions of cells, such as lin^neg^CD38^neg^ cells, the relative rarity of these populations implies that the absolute number of LSCs may be higher in other cell fractions (37). In addition, the incubation of leukemia cells with antibodies targeting surface markers, such as anti-CD38, may reduce the engraftment capacity of leukemia-initiating cells expressing these markers, even further complicating the analysis of human LSCs (37, 38).

In addition to the challenging task of characterizing an LSC phenotype in AML, there is no standard definition for MRD. MRD may well serve as an indicator for the quality of the response to the treatment and may be a prognostic parameter for disease relapse and the choice and effectiveness of post-remission treatment strategy (39). Whereas CR is conventionally defined by pathologists as the absence (≤5%) of blasts in the BM, the establishment of a definition for MRD is much more difficult. First, a significant proportion of AML patients lack molecular markers, such as FLT3-ITD, NPMmut, or chromosomal translocations that would allow monitoring MRD by molecular methods after induction chemotherapy. Second, the time point at which patients should first be tested for MRD and the time interval of serial monitoring is controversially discussed (40). Feller et al. suggested an interval of 3 months for MRD testing by flow cytometry (41). Third, the best method to quantify MRD is still a matter of debate. At the moment, real-time RT-PCR for molecular markers and immunophenotype using multi-parameter flow cytometry are comparable in terms of sensitivity and specificity; however, therapy-related changes in these parameters may limit the clinical applicability (42). Fourth, the level of transcript as measured by RT-PCR or number of cells as measured by flow cytometry defining the threshold for MRD^+^ vs. MRD^−^ has to be validated in prospective studies. And last, the question remains whether peripheral blood can replace BM as the source of cells, which is a relative prerequisite for the feasibility of such studies (39, 40). In summary, all these questions should be addressed during the design of future studies on MRD therapy.

## Myeloid Leukemias and the Immune System

Clinical and experimental observations suggested that myeloid leukemias are partly controlled by the immune system (43). Leukemia cells express major histocompatibility class (MHC)-I and -II molecules and co-stimulatory ligands, such as CD80 and CD86, and therefore may be recognized by T cells and induce potent T cell responses (44–48). In addition, myeloid leukemias respond to unspecific immune-mediated therapies such as IFN-α and interleukin (IL)-2 (49, 50). Furthermore, aHSCT, a treatment with proven ability to cure myeloid leukemias, is in fact an immunotherapy exploiting the allogeneic T and NK cell-mediated GvL effect, which is absent in syngeneic HSCT (22, 51, 52). In addition, it was shown that patients receiving T cell depleted aHSCT grafts had a greater risk of disease relapse, and DLIs from original donors led to CR in most of the patients suffering from disease relapse (53–56).

An interesting example of endogenous immunosurveillance was observed in non-transplanted pediatric AML patients. Montagna et al. demonstrated that stable remission after cytotoxic chemotherapy was associated with the emergence of leukemia-specific CTL precursors in the BM. All patients that had high numbers of CTL precursors remained in remission, whereas the majority of patients with no CTL precursors relapsed (57).

Leukemic cells can be controlled either via specific major histocompatibility complex (MHC-restricted) mechanisms or via less specific incompatibilities in minor histocompatibility genes (58). Indeed, CTLs directed against leukemia antigens have been detected in the peripheral blood of chronic phase CML patients (59, 60) and have been shown to kill CML target cells *in vitro* via Fas-receptor triggering (61). Similar anti-leukemic CTL responses have also been documented in AML (62). In contrast, blast crisis CML cells are refractory to Fas-ligand induced apoptosis, regardless of the expression levels of Fas-receptor, suggesting that an immune-mediated selection by CTLs could result in the acquisition of Fas resistance (63). A further line of evidence that CML is controlled by CTLs comes from our own studies in a murine CML model using the glycoprotein of lymphocytic choriomeningitis virus (LCMV) as a model tumor-antigen. CML-specific CTLs were present in CML-bearing mice and displayed an exhausted phenotype as analyzed by low cytotoxicity, absence of IFN-γ and TNF-α production and expression of programed death-1 (PD-1). Nevertheless, these CTLs contributed to disease control, as depletion of CD8^+^ T cells led to rapid disease progression and death (64). We documented that leukemia-specific CTLs are able to interact with and kill CML LSCs *in vitro* and *in vivo* in a setting with minimal leukemia load. In contrast, in a clinically relevant setting of high leukemia load, CTLs did not kill LSCs but promoted their proliferation by secreting high amounts of IFN-γ (65). In addition, we demonstrated that CD70-expressing T cells stimulate CD27-expressing LSCs in a cell-contact-dependent manner: ligation of CD27 on LSCs by CD70 on T cells reinforced the Wnt-pathway in LSCs, leading to LSC proliferation and disease progression (66). Thus, as it has been shown for other tumors, the immune system interacts with leukemia (stem) cells and may as well play a paradoxical role in promoting disease progression (67).

The role of CD4^+^ T cells in the control of CML has been studied less intensively (68). CD4^+^ T cells isolated from the BM of CML patients were able to suppress autologous hematopoietic progenitor cells in a contact-dependent manner (69). DLIs, depleted of CD8^+^ T cells to reduce the side effects of GvHD, were able to induce remissions in aHSCT-treated CML patients after disease relapse. This led to the hypothesis that CD4^+^ T cells are the main effectors of the GVL effect, whereas CD8^+^ T cells are mainly responsible for GVHD (70). Endogenous CD4^+^ T cells, however, might be dysfunctional *in vivo*, as they have a normal intrinsic cytokine-producing ability only *in vitro*, but not in the leukemia environment (71). However, CD4^+^ T cells may be important in the setting of aHSCT. CD8^+^ T cell-depleted DLI, administered to patients after aHSCT, induced a low rate of remissions and of GvHD (70). Therefore, CD4^+^ T cells are also potentially involved in the GvL effect in CML patients. On the other hand, CD8^+^ T cells may serve as important effectors of GvHD without being essential for GvL.

The roles of B cells and NK cells in the control of CML remain controversial. BCR/ABL1 junctional peptides could induce production of specific antibodies to BCR/ABL1 (72). In addition, it was noted that CD4^+^ DLIs increased the numbers of circulating B cells in patients at the time of clinical response (73). Although antibodies recognizing many distinct leukemia antigens were discovered (74), the impact of antibodies on malignant CML cells remains elusive. NK cells were shown to selectively lyse CML progenitor cells *in vitro* (75). In accelerated CML and blast crisis, NK cell frequency, proliferation, and lytic function seems to decline, but it is currently unclear whether this decline is a cause rather than an effect of disease progression (76, 77). Moreover, donor-vs.-recipient NK cell alloreactivity could eliminate leukemia in human transplants (78).

Chronic myeloid leukemia patients have significantly reduced numbers of circulating myeloid and plasmacytoid DCs (pDCs) compared to healthy volunteers (79, 80). However, BCR/ABL1-expressing DCs have been detected in the peripheral blood of CML patients suggesting that CML derived DCs may possibly contribute to anti-leukemic immunity (81, 82). BCR/ABL1-expressing DCs could be generated from peripheral blood mononuclear cells (PBMCs) or CD34^+^ progenitor cells of CML patients and were shown to have an impaired capacity to capture and process antigens and an impaired migratory capacity compared to DCs derived from healthy controls (83–85). In addition, leukemic DCs were shown to produce TNF-α and IL-8 (86). However, contradictory results about the maturation status of BCR/ABL1 DCs have been published (81, 82).

In summary, it seems plausible that innate as well as adaptive immunity play an important role in the control of myeloid leukemias.

### Immune evasion mechanisms

Myeloid leukemias employ several strategies to compromise anti-leukemic immune responses. DCs originating from myeloid leukemia progenitor cells have been found *in vivo* in leukemia patients and were shown to be abnormal in numbers and function (80–82, 87). Leukemia-derived DCs (L-DCs) displayed reduced antigen-capture and processing capacity, a low maturation status and an aberrant homing pattern when compared to normal DCs (86, 88). Furthermore, L-DCs promoted T cell anergy and the generation of regulatory T (T_reg_) cells instead of inducing CTLs (89–91). T_regs_ are increased in myeloid leukemias (92, 93), are associated with an unfavorable outcome (94), correlate with disease relapse after aHSCT (95) and impede the function of adoptively transferred CTLs (96). Leukemic blasts express high levels of co-inhibitory molecules and interact poorly with T cells due to an impaired formation of immune synapse (97, 98). AML and CML cells for example express the ligands for programed death-1 (PD-L1, PD-L2), which interact with PD-1 expressed on T cells (64, 65, 99, 100). Accordingly, we recently demonstrated that CML LSCs express PD-L1 and PD-L2 as well (65). A further mechanism leukemic cells use to interfere with the immune system is the presentation of MHC class II-associated invariant chain-derived peptide (CLIP). CLIP expression on AML blasts predicts poor clinical outcome (101) and disturbs the activation of leukemia-specific CD4^+^ T cells (102), most probably by interfering with the loading and presentation of LAAs (103). Interestingly, CLIP could also promiscuously bind to various MHC class I types in leukemia cells deficient of MHC class II, a feature that could hamper CTL-mediated leukemia immunosurveillance (104).

The role of tumor necrosis factor (TNF) and TNF-receptor superfamily members in the pathophysiology of leukemia has recently been documented. Glucocorticoid-induced TNFR-related protein ligand (GITRL) was shown to be expressed in a majority of AML cell lines and blasts from patient samples. Reverse signaling of GITRL in AML cells induced the release of TNF and IL-10, and triggering of GITR expressed on NK cells impaired NK cell cytotoxic function and IFN-γ production (105). AML cells exploit further signaling axes of the TNF/TNFR superfamily, such as the 4-1BB-ligand/4-1BB (CD137L-CD137) pathway and the receptor activator of nuclear factor kappa B (RANK)-ligand RANK pathway (106, 107) to inhibit the immune system in a similar way as described for GITR. Furthermore, we could recently document a role for the TNFR CD27 on proliferation of CML LSCs and CML progression (66). Blocking inhibitory pathways holds promise for clinical development. Among them are FAS-ligand that induces apoptosis of FAS-expressing T cells, CD200 that directly inhibits T and NK cells, reactive oxygen species (ROS) that induce lymphocyte apoptosis, killer-cell immunoglobulin-like receptors (KIR) that suppress NK cells and indoleamine 2,3-dioxygenase (IDO) that depletes tryptophan required for T cell expansion or IL-10 that potently suppresses T cell activation [reviewed in Ref. (27)]. Besides inhibiting the adaptive immune system, it was recently demonstrated that leukemic cells are able to block programed cell removal by innate immune cells, thereby overcoming a further regulatory mechanism that normally limits cancer growth. The up-regulation of so-called “don’t eat me” signals on blasts and leukemia stem cells (LSC), such as CD47 and CD200, precludes apoptosis-independent phagocytosis by macrophages. In addition to enable the propagation of the malignant cells, this mechanism likely allows metastatic circulating cancer cells to survive in niches rich in phagocytes, such as the spleen and lymph nodes (108, 109).

These and further immunosuppressive mechanisms remain major hurdles to be overcome in order to successfully implement DC-based immunotherapy in the treatment of leukemia. Interfering with negative immune regulators may effectively improve DC-based immunotherapy, as has been shown by silencing the suppressor of cytokine signaling 1 (SOCS1) or the immunosuppressive cytokine IL-10, which enhanced antigen-presentation and secretion of IL-12 by DCs and triggered an effective anti-tumoral immune response (110–112).

### Cross-priming of CTLs by DCs

Cross-presentation is fundamental for the maintenance of peripheral tolerance and the induction of cross-priming. The concept of cross-presentation defines the processes of antigen uptake and processing and presentation on MHC class I by professional APCs to CTLs (113, 114), whereas cross-priming describes the stimulation and activation of naïve CTLs by this process (115). According to our current understanding that is primarily derived from viral infection models, CTL cross-priming takes place in secondary lymphoid organs (116). Antigen-experienced, matured DCs migrate and transport the viral antigen from the infection site for cross-presentation to secondary lymphoid structures (117).

The crucial factor for DCs to tune CTL activation is their maturation status (118). Several studies demonstrated that the presence of appropriate inflammatory and co-stimulatory maturation signals, such as pathogen-associated molecular patterns (PAMPs), TLR ligands, type I IFNs, CD80/CD86, and CD70 (119, 120) as well as CD40 ligand (CD154) provided by CD4^+^ T cells (“DC licensing”) is essential for DCs to properly activate CTLs in viral infections (118). It is well documented that solid and hematological tumor microenvironments contain DCs in mice and men [reviewed in Ref. (121)]. These microenvironments, however, lack DC-activating and DC migration-inducing factors (122) and harbors a multitude of immunosuppressive molecules such as TGFβ and IL-10 that impair DC maturation, migration and antigen (cross-) presentation [reviewed in Ref. (123)]. AML blasts can generate an immunosuppressive microenvironment that hinders effective adaptive as well as innate immune responses (124–127), such as by the secretion of arginase II resulting in T cell inhibition (124). Cross-presentation of the LAAs proteinase-3 and PR1 has also been shown in AML patients, but these antigens were presented by immature DCs resulting in tolerization of CTLs (128).

Therefore, even though there is compelling evidence that LAAs are cross-presented *in vitro* and *in vivo*, the question as to what extent the process of cross-priming contributes to anti-leukemic immunity is still highly controversial (114).

Nevertheless, fully functional CTLs are fundamental for the surveillance, control, and elimination of tumors (129, 130). Therefore, a better understanding of specific DC subsets in the anti-leukemic immune response and how cross-presentation of LAAs *in vivo* can be improved and consequently CTL dysfunction circumvented, may lead to improved vaccine-based immunotherapy against leukemia.

## Leukemia Antigens

In order to achieve an optimal and effective immune response with a low rate of toxicity, leukemia antigens that are specifically expressed and presented by leukemia cells and not by healthy tissue have to be identified. In addition, these should be immunogenic and should critically account for the leukemic phenotype. Most importantly, however, these antigens should be expressed in LSCs, even though currently the phenotypic characterization of LSCs is controversial and elusive. The restricted numbers of clearly identified LAAs in leukemia remain a major obstacle for the use of these peptides in DC-based immunotherapy. In addition, the low affinity of these LAAs to bind MHC I, the short time of antigen-presentation on DCs as well as the lack of help by CD4^+^ T cells may limit the capacity of these LAAs to induce an anti-tumoral immune response (131, 132).

The most specific leukemia antigens are peptides from aberrant proteins created by mutations or translocations only present in leukemia cells, such as the BCR/ABL1 tyrosine kinase in CML. These peptides are known as leukemia-specific antigens (LSAs). However, most of the leukemia-specific mutations and translocations do not give rise to proteins (133). Among the numerous chromosomal translocation that were characterized in AML, only a minor fraction such as AML1-ETO (133), DEK-CAN (134), and PML-RARα (135) gives rise to proteins that generate LSAs. In addition, only two mutations involving the fms-related tyrosine kinase (FLT) and nucleophosmin 1 (NPM1) have been shown to give rise to LSAs (136, 137). Therefore, most immunotherapy approaches in myeloid leukemia target LAAs, that is, peptides from proteins that are expressed in leukemic cells and also healthy tissues, but are often overexpressed in leukemia and important for the malignant phenotype. Consequently, the induction of autoimmunity is a potential risk if such LAAs are chosen as targets for an immunotherapy. As an additional limitation, T cell receptors recognizing antigens that are broadly expressed on healthy tissues in the body are usually of low affinity. Therefore, it is crucial to characterize the degree of LAA expression on normal tissues in order to envisage the multitude and characteristics of potential autoimmune reactions.

For AML, a multitude of LAAs has been described during the last two decades and has been validated as target for immunotherapy [Table 1 and reviewed in Ref. (133)]. These LAAs comprise proteinase-3, Wilms tumor protein (WT1) (62, 138–141), the receptor for hyaluronic acid-mediated motility [RHAMM/CD168 (142)] human telomerase reverse transcriptase [hTert (143)], preferentially expressed antigen in melanoma [PRAME (144, 145)], and Aurora-A kinase (146) (Table 1).

**Table 1:** **Leukemia-associated antigens (LAAs) in myeloid leukemias**.

Myeloid leukemia	LAA	Reference
AML	Aurora-A kinase	(146, 153, 154)
	BRAP	(160)
	Cyclin A1	(161)
	hTert	(143)
	HSJ2	(160)
	MPP11	(160)
	Neutrophil elastase (NE)	(166)
	PRAME	(144, 145, 162)
	PR1	(128, 139, 163, 164)
	Proteinase-3	(62, 164, 165)
	RBPJκ	(160)
	RHAMM/CD168	(142)
	WT1	(62, 139, 141, 148, 149, 151, 152)
CML	BRAP	(160)
	CML-28	(167–169)
	CML-66	(167–169)
	HAGE	(168)
	HSJ2	(160)
	MPP11	(160)
	PRAME	(144)
	PR1	(59, 139, 164, 169)
	Proteinase-3	(164, 165, 169)
	RBPJκ	(160)
	Survivin	(167–169)
	WT1	(139, 148, 149, 169–171)

*AML, acute myeloid leukemia; BRAP, BRCA1-associated protein; CML, chronic myeloid leukemia; HAGE, helicase antigen; HSJ2, heat-shock 40 kDa protein 4; hTert, human telomerase reverse transcriptase; MPP11, M-phase phosphoprotein 11; PRAME, preferentially expressed antigen in melanoma; RBPJκ, recombination signal binding protein for immunoglobulin kappa J region; RHAMM, receptor for hyaluronic acid-mediated motility; WT1, Wilms tumor protein*.

The most attractive and promising LAA is the tumor-suppressor gene and zinc finger transcription factor WT1. WT1 is a regulator of cell proliferation, differentiation, and apoptosis. In leukemia, WT1 has been shown to have a fundamental oncogenic role for leukemogenesis resulting in differentiation arrest and aberrant cell growth (147). WT1 was demonstrated to be immunogenic as it elicits a naturally occurring anti-tumoral immune response in cancer patients (148, 149). In addition, in a WT1 directed immunotherapeutic study, off-target toxicity effects have not been observed, indicating that WT1-expressing normal tissues are omitted from the response (150). However, in some AML patients no WT1-specific CTL response has been triggered even though objective responses and remissions have been elicited (141). Importantly, WT1 is expressed to a much lesser extent on normal HSCs than on leukemic blasts and LSCs in a majority of AML patients which characterizes WT1 as attractive target for immunotherapy in AML (151, 152). Consequently, WT1 is currently targeted in clinical T cell therapy and vaccination studies.

Importantly, in a curative approach LAAs have to be expressed on LSCs (146, 153, 154). One LAA in AML that is expressed on CD34^+^CD38^−^ AML “stem” cells compared to CD34^+^CD38^+^ AML progenitor cells and normal CD34^+^ stem/progenitor cells from healthy individuals is the serine/threonine kinase Aurora-A. Importantly, CD34^+^ leukemia progenitor cells but not normal CD34^+^ stem/progenitor cells were lysed by Aurora-A kinase-specific CTLs. Furthermore, blockade of Aurora-A kinase by a small-molecule inhibitors or shRNA impaired engraftment and improved survival of mice in an AML xenograft model (146, 153, 154).

In CML patients numerous LAAs such as WT1, proteinase-3, cancer-testis antigens like HAGE, minor histocompatibility antigens, hTert, CML-66, CML-28, and survivin were shown to be aberrantly expressed in the transformed CML cell (Table 1). Some LAAs such as hTert and survivin have a quite restricted expression pattern and are not or only marginally expressed on normal non-dividing or terminally differentiated cells (143, 155). This makes hTert and survivin promising targets for DC-based immunotherapy.

The most prominent LSA in CML is the chimeric BCR/ABL1 fusion protein, an ideal target for immunotherapy (8). An elegant paper by Yotnda et al. identified a BCR/ABL1 junctional nonapeptide (SSKALQRPV) that binds to human leukocyte antigen (HLA)-A2.1 and elicits specific CTL responses *in vitro* in blood from healthy donors and CML patients. In 5 out of 21 CML patients, the investigators found high frequencies of junctional peptide-specific CTLs in the peripheral blood, suggesting an *in vivo*-immunogenicity of this peptide (156). Additional studies confirmed and extended the finding of immunogenic BCR/ABL1 junction peptides (157, 158). However, BCR/ABL1 is gives rise to a limited number of immunogenic epitopes due to only two chromosomal breakpoints (159). Furthermore the expression of the epitopes is restricted to HLA A2, A3, and B7 (158).

Since all the LAAs listed in Table 1 are expressed to a greater extent on malignant cells than on their healthy counterparts, they represent suitable antigens for immunotherapeutic approaches.

## Immunotherapy for Myeloid Leukemias

Nowadays, immunotherapy covers a huge spectrum of distinct experimental procedures in order to specifically eliminate cancer cells while minimizing harm to normal tissue to limit side effects (172). However, up to now only few approaches have entered clinical routine such as unspecific immune stimulation by Bacillus Calmette–Guérin (BCG) instillations to treat non-muscle invasive bladder cancer after surgical ablation (173) or the immunomodulating anti-CTLA4 mAb Ipilimumab for metastatic melanoma or prostate cancer (174), as well as aHSCT for the treatment of myeloid leukemias (175) and the prostate antigen-specific DC-based vaccine Sipuleucel-T (Provenge^®^) for hormone-refractory prostate cancer (176).

The intention of active cancer immunotherapy is to mount an endogenous adaptive immune response against a tumor by directly injecting tumor-antigens together with adjuvants (“peptide vaccines”) or by *ex vivo*-generation of cancer-specific DCs (“DC vaccines”) and to form CTL memory in order to sustain remission (177). For AML and CML, numerous studies extensively investigated the clinical potential of this approach. Administration of autologous DCs loaded via electroporation with mRNA of the LAA WT1 resulted in CR in 50% of AML patients in a phase I/II study (141). Importantly, CR was achieved in two patients that only had partial remission after chemotherapy, indicating the feasibility and clinical potential of this approach. In contrast, in a clinical phase I study, autologous monocyte-derived DCs (mDCs) previously cultured in the presence of AML failed to induce a clinical response in relapsed AML patients (178).

Recently, a better understanding of immunosurveillance paved the way for the development of new immunotherapeutic approaches and their implementation in the clinics. Among these, immune checkpoint inhibition is most advanced in melanoma patients and anti-CTLA4 blockade was actually the first therapy that improved survival of patients suffering from metastatic melanoma (174). A recent hallmark immunotherapeutic study using a dual mAb treatment approach to block the immune checkpoint regulators CTLA-4 and programed death-1 (PD-1) using Ipilimumab and Nivolumab, respectively, resulted in persistent tumor regression in advanced melanoma patients (179). AML and CML cells also express the ligands for PD-1 (PD-L1, PD-L2), which interact with PD-1 expressed on T cells (64, 65, 99, 100). Accordingly, we recently demonstrated that CML LSCs express PD-L1 and PD-L2 as well (65). In addition, we recently demonstrated that blocking PD-1 signaling results in improved CML control in pre-clinical mouse models (64).

Furthermore, chimeric antigen receptor (CAR) T cells for adoptive T cell therapy (ACT) proved their clinical potential in leukemia patients. In addition, ACT with CAR T cells overcame the obstacle to generate sufficient numbers of high avidity LAA-specific T cells *in vitro* and long-term persistence, memory formation, and migration *in vivo*. Chronic lymphocytic leukemia (CLL) patients treated with a low number of CAR T cells targeting CD19 and containing the co-stimulatory signaling domain of CD137 exhibited a CR. Importantly, CAR T cells extensively expanded and showed a CD19-specific immune response as well as long-term persistence with an effector memory phenotype in peripheral blood and BM without the need to trigger an anti-leukemic immune response by professional APCs. This phenotype consequently allows potential expansion upon secondary encounter with CLL cells and prevention of relapse (180). Furthermore, two children with relapsed and refractory pre-B cell ALL treated with CD19-specific CAR T cells were reported to have achieved CR (181). For myeloid leukemias a clinical application of CAR T cells has not yet been documented. However, CAR T cells targeting isoform 6 of CD44 (CD44v6) that is expressed by AML cells (182) but not by HSCs and at low levels on normal cells (183) mediated potent anti-tumor effects against primary AML in a pre-clinical AML model (184). In addition, clinical phase I/II studies (NCT01640301, NCT01621724) using ACT of T cells carrying a TCR specific for the LAA WT1 in AML patients are ongoing. These trials are essential to further determine if safety and efficacy of this promising immunotherapeutic approach also holds true for the treatment of AML patients.

### DC-based immunotherapy in leukemia

Because of their excellent ability to activate T cells, DCs are considered as one of the most promising tools for tumor-antigen delivery in active cancer immunotherapy and they are ideal candidates to supply foreign tumor-antigen in the form of a DC-based vaccine or for the generation of tumor-antigen-specific CTLs *in vitro* (185). Clinical studies have used various precursor cells in order to manufacture sufficient *ex vivo* tumor-antigen loaded DCs for immunotherapeutic purposes (186). However, the different methods in manufacturing those DCs and the notion that the generated DCs differed in function and phenotype resulted in need for the standardization of DC vaccines.

To vaccinate AML patients with DCs in order to induce an optimal, long-lasting anti-leukemic CTL response, several issues have to be considered:

First, the type and origin of DCs used to treat the patient has to be defined. DCs can either be generated from patient-derived CD34^+^ cells or CD14^+^ monocytes *in vitro*. They can be directly positively selected from the patient’s PBMCs (*ex vivo*) and are differentiated in the presence of various cytokines which improves the LAA loading onto these DCs (177). Additionally, naturally circulating DCs can be loaded and activated *in vivo* using mAbs targeting SIGLEC H conjugated to an LAA in combination with CpG nucleotides (187, 188). In leukemia patients, especially in AML patients, the presence of blast-derived leukemic DCs was extensively documented (80–82, 87). Consequently, a promising method of generating L-DCs is to differentiate blasts from AML patients into DCs *ex vivo*. This method allows circumventing the loading of the DCs with LAAs. The application of these AML-derived DCs in a clinical setting is still poorly developed. Especially, the generation of sufficient numbers of AML-derived DCs is challenging. Only 25% of the initial AML cells cultured can be converted into AML-derived DCs. In addition, AML-derived DCs can only be generated in around 40% of AML patients due to AML-specific mutations (e.g., Flt-ITD) or the lack of CD14 expression that prevent the conversion of blasts into AML-derived DCs (189, 190). Nonetheless, the tolerability of this therapeutic approach and the induction of an anti-leukemic immune response in patients have been already reported. Despite these positive reports, the clinical benefit of the DC vaccine is only marginal (191). Therefore, the current DC-based cancer immunotherapy protocols using AML-derived DCs are optimized and standardized in order to allow generating sufficient AML-derived DCs (192, 193) with an improved potential to prime and activate CTLs and increase their cytolytic capacity (194, 195).

The other critical factors determining the success of DCs in AML immunotherapy besides the generation of sufficient numbers of DCs are (1) the selection of the proper LAA (discussed later), (2) the method applied for loading the respective leukemia antigen onto the DCs, and (3) the strong activation of DCs necessary to provide sufficient co-stimulatory signal for efficient T cell activation and to prevent T cell tolerization.

**Figure 3:**
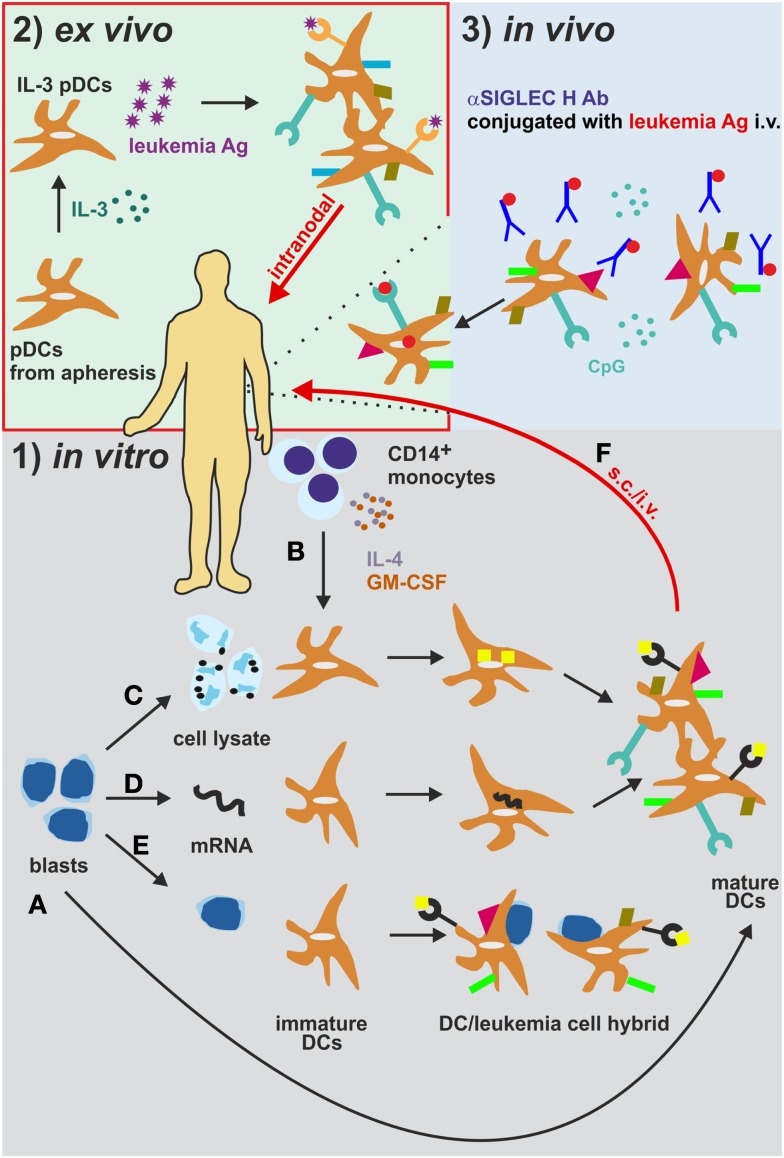
**Different strategies for the generation and administration of DC-based vaccines in AML**. (1) **(A)** Leukemia-derived DCs can be directly generated by isolation and differentiation from AML blasts *in vitro*. **(B)** CD14^+^ monocytes from patients or healthy donors are differentiated into monocyte-derived DCs (mDCs). These mDCs are cultured together with **(C)** AML cell lysates or immunogenic apoptotic/necrotic AML cells (185) or **(D)** are electroporated with mRNA from AML cells (191) to ensure leukemia antigen loading. **(E)** As an additional *in vitro* approach, AML blast-mDC cell-fusion hybrids are artificially generated (196). **(F)** The DCs are then injected s.c. or i.v. into AML patients. (2) DCs can also be loaded and activated *in vivo* (188). DCs express the endocytosis receptor SIGLEC. Intravenous administration of an αSIGLEC H mAb conjugated to a leukemia antigen in the presence of CpG results in DC activation, antigen uptake and presentation. (3) Plasmacytoid DCs isolated from AML patients are activated and loaded with leukemia antigens *ex vivo* and are re-injected intralymphatically into lymph nodes (201). Ab, antibody; Ag, antigen; i.v. intravenously; pDCs, plasmacytoid DCs. s.c., subcutaneously.

Originally, mDCs have been cultured together with AML cell lysates or immunogenic apoptotic/necrotic AML cells to ensure LAA loading [Figure 3 (185)]. As an additional approach, AML blast-mDC cell-fusion hybrids have been generated *in vitro* [Figure 3 (196)]. Importantly, all these multi-epitope approaches might deliver a variety of known and unknown LAAs to the DCs. In addition, these approaches circumvent the need for previous identification of the LAAs. On the other hand, co-culturing or fusion approaches might negatively impact the antigen uptake and processing and/or the maturation of DCs because of immunosuppressive factors stored in or produced by AML cells, such as TGF-β (185). Nevertheless, Herr et al. have shown that tumor cell lysate-loaded DCs were superior to DCs loaded with eluted peptides in inducing an anti-tumoral immune response against an EBV^+^ B lymphoblastic cell line (197). Nowadays, pre-clinical and clinical approaches favor the loading of DCs with peptides from LAAs or LSAs such as WT1, Survivin, PML-RARα, etc. via peptide pulsing or electroporation and mRNA loading [Figure 3 (191)]. Most of the studies using one of these loading methods reported activation and expansion of HLA-compatible CTLs *in vitro* resulting in killing and eliminating of the leukemia cells, indicating a reasonable rationale to apply mDC immunotherapy in a clinical setting irrespective of the antigen loading method. However, the use of single antigens poses the risk of immunoediting and the escape of antigen-loss variants (198). Especially, the technique of mRNA electroporation offers several advantages to overcome this issue: (1) simultaneous loading and presentation of multiple LAA epitopes without any risk for insertional mutagenesis due to the only transient mRNA expression (199); (2) expression of multiple patient-specific LAAs at once, when electroporation is performed with whole AML cell lysate mRNA (200); and (3) the possibility of combination with other loading methods (200).

For optimal DC activation and antigen processing of *in vitro* generated DCs, different cocktails of cytokines and TLR ligands have been tested. Usually, patient-derived monocytes are cultured in the presence of IL-4 and granulocyte-monocyte colony stimulating factor (GM-CSF) for several days, followed by a short course of DC maturation using TLRs, pro-inflammatory cytokines such as TNF-α, IL-1β, IL-6, prostaglandin E2, and/or CD40 ligand (202). Similar procedures have been applied for AML blast-derived DCs (185, 203–210). However, the effects of these *in vitro* culture and maturation conditions on the ability of DCs to capture, process and present antigen, on their T cell activating potential and on their *in vivo* migratory function are not fully understood (177). For example, the replacement of IL-4 by IL-15 during the differentiation phase was shown to enhance the immunostimulatory properties of DCs with a phenotype and characteristics of Langerhans cells (LCs), which are *per se* far more efficient in antigen-presentation and T cell priming *in vitro*. (211, 212). In addition, it was demonstrated that DCs matured conventionally in the presence of pro-inflammatory cytokines are unable to produce IL-12 *in vivo*, a cytokine that is essential for CD4^+^ T_H_1 cells differentiation. Maturation in the presence of the TLR7/8 agonist R848 restored IL-12 secretion, improved cell migration and led to more robust induction of anti-leukemic immune responses *in vitro* (202, 213, 214).

Tracking of labeled and intradermally administered DCs in patients revealed that less than 1% of the DCs are migrating into the adjacent lymph nodes (215). In order to circumvent the drawbacks of *in vitro* DC generation and their poor migration into lymph nodes, in a clinical study of DC-based immunotherapy in melanoma, Tel and colleagues directly isolated human pDCs and injected them intralymphatically into the inguinal lymph nodes after *ex vivo* activation and loading (201). pDCs, specialized DCs that are characterized by rapid and massive secretion of type I IFNs in response to foreign nucleic acids, have been shown to successfully mediate an interplay of innate and adaptive immune responses by activating other DCs and inducing cross-priming (216–218). Compared to subcutaneous injections, intralymphatic immunotherapy substantially reduces the amount of vaccine necessary and the duration of immunization. This approach has already proven effective for the treatment of allergies [Figure 3 (219)]. Therefore, pDCs and/or intralymphatic injection protocols may become crucial players in eliciting anti-leukemic immunity.

**Figure 4:**
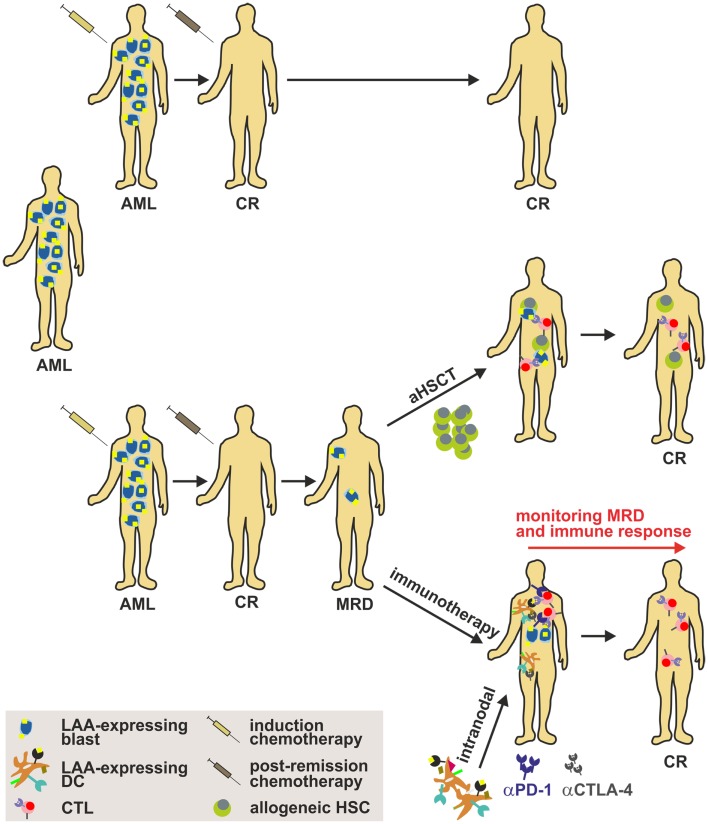
**Strategy to implement DC-based immunotherapy in the treatment of AML**. Induction cytotoxic chemotherapy (“3 + 7” scheme with cytarabin and an anthracycline) results in a labile complete remission (CR) that has to be consolidated by post-remission chemotherapy. Nonetheless, many patients harbor persistent LSCs after a CR (referred to as MRD), which may cause disease relapse. Therefore, strategies such as aHSCT (only for a minor fraction of patients) or immunotherapy have to be implemented to sustain CR. Importantly, DC-based immunotherapy targeting AML-specific LAAs alone or in combination with immune checkpoint inhibitors such as anti-CTLA-4 or anti-PD-1 mAbs might be a promising approach to treat patients and to target and eliminate LSCs. aHSCT, allogeneic hematopoietic stem cell transplantation; AML, acute myeloid leukemia; CTL, cytotoxic lymphocyte; CR, complete response; DC, dendritic cell; LAA, leukemia-associated antigen; MRD, minimal residual disease.

By now, it is unfortunately not fully elaborated which DC subset is most suitable for DC-based immunotherapy. The identification of this subset, the optimal route of administration, the optimal dose, the optimal antigen, and conditioning in order to maximize the efficacy of the treatment is pivotal for the success of treatment. Therefore, future studies have to fully aim at the functional characterization of different DC subsets in terms of T cell (cross-) priming, migration capacity, cytokine production, half-life etc. in order to maximize the clinical benefit of the therapy. The fundamental challenge in the treatment of AML remains the prevention of clinical relapse of the patients. The generation of clinical grade AML-derived DCs from AML patients in remission has been reported (220) and may consequently serve as a potential strategy in order to avoid a potential relapse (Figure 4). In addition, results from recent clinical phase I/II studies treating AML patients in remission with clinical grade DCs generated with different protocols highlight the importance of the selection of the antigen, the loading approach as well as the time of administration as fundamental success criteria for DC-based immunotherapy in AML.

## Clinical Trials

Currently, several peptides derived from LAAs are under clinical investigation for myeloid leukemia patients in current vaccination trials. Ongoing or recruiting DC vaccination trials in phase I and II use either different WT1 derived peptides (NCT01686334, NCT00834002, NCT00672152, NCT01266083), the proteinase-3-derived peptide PR1 (NCT00454168), the peptides MAGE-A1, MAGE-A3, and NY-ESO-1 (NCT01483274) or a combination of WT1 and PR1 (NCT00433745, NCT00488592). These trials primarily include patients that just underwent aHSCT, elderly patients or patients in first remission. Interestingly, one study that has been completed recently applied a vaccination protocol with lethally irradiated autologous AML cells that were genetically modified to secrete human GM-CSF in order to enhance LAA presentation (NCT00136422). Another trial that aimed at up-regulating LAA presentation additionally administered the hypomethylating drug decitabine (NCT01483274). More and more studies use DC vaccination in combination with other drugs or cytokines. For example, in a clinical phase II study, CML patients in remission are treated with PR1 peptide vaccine in combination with pegylated IFN-α2b (PegIntron^®^, NCT00415857). Another approach combines a DC cell/AML fusion vaccine with the blockade of PD-1 (NCT01096602).

All these clinical trials have proven that DC-based immunotherapies in leukemia are safe and have hardly any side effects. Unfortunately, this good tolerability is accompanied by a rather minor clinical benefit in terms of response rate or other important clinical outcome parameters (191). From immunotherapy trials in solid tumors we have learned that the established response criteria for chemotherapy, such as the “RECIST criteria,” may not be appropriate for immunotherapy approaches. This may also hold true for leukemia. Reduction of leukemia load or remission in the BM shortly after the treatment may not be the appropriate readout to judge the efficacy of an active immunotherapy that needs time to be established and may contribute to a long-term control of the disease. In addition, suitable biomarkers that are predictive for a response to an immunotherapy are still lacking (177). Therefore, future studies also have to focus on the generation of adequate readouts and the identification of defined biomarkers for the monitoring of DC-based immunotherapy in leukemia. Furthermore, most clinical studies carried out so far enrolled leukemia patients with a high leukemia burden. In these studies, at least some of the patients showed a minor clinical benefit. Importantly, applying DC-based immunotherapy to patients with a lower leukemia burden or MRD might result in better responses.

## Concluding Remarks

During the last decade, the combined efforts of researchers to treat myeloid leukemia unraveled a multitude of LAAs suitable for DC-based immunotherapy. Consequently, DC-based immunotherapy slowly progresses into the clinical treatment of leukemia.

The rapid development in the field allowed the design of phase I and II studies with different DC vaccination protocols. DC-based vaccination often resulted in the induction of potent anti-leukemic CTL responses. The benefit for the patient in these studies in terms of response to treatment was rather limited. Nevertheless, DC vaccination protocols remain a promising supplementary strategy in the treatment of leukemia, and future improvements will reveal their full potential. In order to improve DC-based vaccination for clinical routine, several issues still have to be solved. Most importantly, an optimal timing for the vaccination during the course of disease has to be defined. Current literature and our own experiments indicate that immunotherapy may be most effective in the state of MRD after successful induction and post-remission chemotherapy. In parallel, MRD has to be better defined, characterized, and especially quantified by the introduction of more sophisticated molecular and flow cytometry techniques. Simultaneously, it is of extreme importance to quantitatively and functionally assess the degree of the anti-leukemic CTL response. Furthermore, the vaccination procedure, including the choice of LAA (or multiple LAAs); the source of DCs (mDCs, LCs, pDCs, or AML-derived DCs); the DC maturation protocol and the way of application (i.v. vs. s.c. vs. intralymphatical) have to be defined and standardized. Finally, the timing and application of potential co-treatments, including chemotherapy, aHSCT and immunomodulating agents has to be considered. Especially, combining DC-based immunotherapy with the blockade of immune checkpoint regulators such as PD-1 and/or CTLA-4 may represent a powerful tool for the treatment of leukemia.

## Conflict of Interest Statement

The authors declare that the research was conducted in the absence of any commercial or financial relationships that could be construed as a potential conflict of interest.
